# Retinoic Acid Receptor Alpha Is Essential in Postnatal Sertoli Cells but Not in Germ Cells

**DOI:** 10.3390/cells11050891

**Published:** 2022-03-04

**Authors:** Diana Condrea, Sirine Souali-Crespo, Betty Féret, Muriel Klopfenstein, Sylvain Faisan, Manuel Mark, Norbert B. Ghyselinck, Nadège Vernet

**Affiliations:** 1 Institut de Génétique et de Biologie Moléculaire et Cellulaire (IGBMC), Département de Génétique Fonctionnelle et Cancer, Centre National de la Recherche Scientifique (CNRS, UMR7104), Institut National de la Santé et de la Recherche Médicale (INSERM U1258), Université de Strasbourg (UNISTRA), 1 rue Laurent Fries, CEDEX, 67404 Illkirch, France; condread@igbmc.fr (D.C.); soualics@igbmc.fr (S.S.-C.); betty2@igbmc.fr (B.F.); tictac@igbmc.fr (M.K.); marek@igbmc.fr (M.M.); norbert@igbmc.fr (N.B.G.); 2 ICube (UMR 7357), Laboratoire des Sciences de l’Ingénieur, de l’Informatique et de l’Imagerie, UNISTRA, CNRS, 300 Boulevard Sébastien Brant, CEDEX, 67412 Illkirch, France; faisan@unistra.fr; 3 Service de Biologie de la Reproduction, Hôpitaux Universitaires de Strasbourg (HUS), 67000 Strasbourg, France

**Keywords:** anti-RARA antibodies for IHC, mouse, retinoic acid, Sertoli cell, seminiferous epithelium, Sox9-CreERT^2^, spermatogenesis, *Stra8-Cre*, synchronization factor, tamoxifen-inducible Cre recombinase, testis

## Abstract

Retinoic acid signaling is indispensable for the completion of spermatogenesis. It is known that loss of retinoic acid nuclear receptor alpha (RARA) induces male sterility due to seminiferous epithelium degeneration. Initial genetic studies established that RARA acts in Sertoli cells, but a recent paper proposed that RARA is also instrumental in germ cells. In the present study, we have re-assessed the function of RARA in germ cells by genetically ablating the *Rara* gene in spermatogonia and their progenies using a cell-specific conditional mutagenesis approach. We show that loss of *Rara* in postnatal male germ cells does not alter the histology of the seminiferous epithelium. Furthermore, RARA-deficient germ cells differentiate normally and give rise to normal, living pups. This establishes that RARA plays no crucial role in germ cells. We also tested whether RARA is required in Sertoli cells during the fetal period or after birth. For this purpose, we deleted the *Rara* gene in Sertoli cells at postnatal day 15 (PN15), i.e., after the onset of the first spermatogenic wave. To do so, we used temporally controlled cell-specific mutagenesis. By comparing the testis phenotypes generated when *Rara* is lost either at PN15 or at embryonic day 13, we show that RARA exerts all of its functions in Sertoli cells not at the fetal stage but from puberty.

## 1. Introduction

Spermatogenesis, the process that allows for the formation of spermatozoa, consists of three distinct phases: (i) the proliferative phase, during which the spermatogonia stem cells divide and differentiate to maintain both germ cell production and stem cell renewal; (ii) the meiotic phase, during which the spermatocytes undergo two consecutive divisions to produce haploid spermatids; and (iii) the spermiogenesis phase, during which the spermatids differentiate into spermatozoa. Occurring within the seminiferous epithelium of the testis, spermatogenesis is supported by somatic Sertoli cells. Both germ cells and Sertoli cells are impacted upon changes in vitamin A metabolism or its signaling pathway ([1,2] and references therein). For instance, vitamin A deficiency in rodents induces an arrest of spermatogonia differentiation, resulting in the progressive depletion of more differentiated germ cells. Additionally, Sertoli cells lose their cyclical changes in morphology and gene expression [3,4]. Importantly, administration of all-*trans* retinoic acid (ATRA) to vitamin A-deficient rodents restores spermatogenesis [4,5], indicating that ATRA is the active metabolite of vitamin A in the testis.

ATRA acts through binding to retinoic acid receptors (RARs; isotypes RARA, RARB, and RARG) [6]. Although the three RARs are expressed in the testis [7,8,9], they are not equally important for spermatogenesis, as inferred from the phenotypic analysis of knockout mice. *Rarb*-knockout males are fertile, with no alteration of spermatogenesis [10,11,12]. In contrast, *Rara*-knockout males display a pathological phenotype characterized by spermatogenic defects and infertility [13,14]. As for *Rarg*, its knockout yields vitamin A deficiency-like testis degeneration, resulting from an arrest of spermatogonia differentiation [15].

To assess RARA functions in spermatogonia, male mice lacking *Rara* in germ cells were generated and analyzed [16]. In these mutants (called *Rara*-cKO), the seminiferous epithelium was found to be severely affected, displaying extensive vacuolation and sloughing of immature germ cells. Surprisingly, an earlier study did not mention vacuolization of the seminiferous epithelium and germ cell sloughing in mutant males lacking all three RAR isotypes in germ cells [17]. In order to reconcile the discrepancies between these two studies, here we have generated mice lacking only RARA in germ cells, carefully ensured that *Rara* gene excision was obtained, and reassessed their testis phenotype. Despite meticulous analysis, we did not find any abnormality, thus contradicting the results published recently [16] but confirming that RARA is fully dispensable in germ cells for their proper differentiation.

We previously showed that selective ablation of the *Rara* gene in Sertoli cells from embryonic day 13.5 (E13.5) onward fully recapitulates the set of abnormalities found in the testis of *Rara*-knockout males [12,18]—i.e., delay in the first spermatogenic cycle; seminiferous epithelium vacuolization; sloughing of immature germ cells; apoptosis of spermatocytes; and failure of spermiation, the process whereby mature spermatids are translocated and released into the lumen of the seminiferous epithelium. This suggests that all functions played by RARA in the testis are Sertoli cell-autonomous [12]. However, the time period in which RARA is essential to allow proper Sertoli cell functioning in the adult is still unknown, because the *Rara* gene was until now invalidated in Sertoli cells either before or just after the onset of their appearance during fetal development [12,18]. To gain insights into this question, we set up and analyzed in the present study a genetic model in which *Rara* gene deletion occurs after birth. We found that loss of RARA in Sertoli cells from postnatal day 15 (PN15) onwards induces all testis abnormalities displayed by *Rara*-knockout males. This finding indicates that RARA-dependent signaling in Sertoli cells is required at the onset of spermatogenesis but not during fetal stages. Together, our results firmly establish that RARA is essential in postnatal Sertoli cells for spermatogenesis, while it appears to be dispensable in germ cells.

## 2. Materials and Methods

### 2.1. Mice and Treatments

Mice of a mixed C57BL/6-129/Sv (50–50%) genetic background were housed in an animal facility licensed by the French Ministry of Agriculture (agreement no. D-67-218-37). All experiments were approved by the local ethical committee (Com’Eth, accreditations APAFIS#28483-2020120115253832) and were supervised by N.B.G., M.M., or N.V., who are qualified in compliance with the European Community guidelines for laboratory animal care and use (2010/63/UE).

Mice bearing L2 alleles of *Rara*, in which exon 6 is flanked by *lox*P sites [19], were bred with transgenic mice, driving the expression of Cre recombinase (i) in germ cells thanks to the *Tg(Stra8-cre)*^1Reb^ transgene [20], hereafter referred to as *Stra8-Cre*^Tg/0^ mice, or (ii) in Sertoli cells thanks to the tamoxifen (TAM)-inducible *Tg(Sox9-cre/ER^T2^)*^1Msan^ transgene [21], hereafter referred to as *Sox9-CreER^T2^* ^Tg/0^ mice. To monitor for efficient gene excision, we also introduced in each case the *Gt(ROSA)26Sor*^tm1(EYFP)Cos^ transgene, hereafter referred to as *R26R-EYFP*^Tg/0^ mice [22]. This reporter directs the expression of EYFP in cells that have experienced Cre-mediated recombination. Mice were genotyped with primer sets as listed in Table 1.

In *Stra8-Cre*^Tg/0^ mice, the recombinase is first expressed at PN3 in a subset of undifferentiated spermatogonia and in all differentiating spermatogonia [20,23]. Both mutant and control males (bearing and free of *Tg(Stra8-cre)*^1Reb^ transgene, respectively) were generated from the same breeding pairs, in the same litters.

In *Sox9-CreER^T2^* ^Tg/0^ mice, the recombinase is expressed in SOX9-positive cells throughout development and adulthood [21], including the postnatal Sertoli cells [24], where it becomes functional only upon TAM treatment. Experimental males (bearing and free of *Tg(Sox9-cre/ER^T2^)*^1Msan^ transgene) were generated from the same breeding pairs, in the same litters. Pups were administered TAM (50 mg/kg body weight) by intraperitoneal injections, three times every second day, between PN5 and PN15. Both those bearing and those free of the transgene received TAM, precluding therefore that the observed abnormalities at later stages are due to this drug [25]. TAM (T5648, Sigma-Aldrich, 38070 Saint-Quentin-Fallavier, France) was dissolved in ethanol at a concentration of 100 mg/mL and further diluted in sunflower oil to a concentration of 10 mg/mL before administration.

### 2.2. Histology, Stage Frequencies, and Synchronization Factor

For histology, testis and cauda epididymis samples were fixed in Bouin’s fluid for 16 h and embedded in paraffin. Histological sections (5 μm thick) were stained with hematoxylin and eosin (HE) or with periodic acid–Schiff (PAS). Testes from at least three mice per genotype were analyzed at each time point.

In the seminiferous epithelium, the different generations of germ cells form cellular associations of fixed composition (called epithelial stages). Twelve epithelial stages (I–XII) can be identified in the mouse [26,27]. The determination of epithelial stages’ frequencies was performed on PAS-stained sections from 3- to 9-month-old mutants and control mice (bearing and free of *Tg(Stra8-cre)*^1Reb^ transgene, respectively). Sections were scanned in a Hamamatsu NanoZoomer 2.0-HT scanner, and 200 to 300 seminiferous tubules per mouse were analyzed using the NDP.view2 software. The identifying features were as follows: stages I–III, small unstained or weakly stained proacrosomal granule; stage IV, acrosomic granule forming an indentation and beginning to flatten; stages V–VI, acrosomic system forming a straight PAS-positive line, with the angle subtended by the acrosome being lower than or equal to 120; stages VII–VIII, acrosome forming a cap that covers more than one-third of the nucleus; stage IX, oblong spermatid nuclei; stage X, elongating spermatid head displaying a sharp angle between its ventral and caudal surface; stage XI, spermatid nucleus thinner, more elongated and stained more intensely; stage XII, presence of meiotic metaphases, meiotic anaphases, and/or secondary spermatocytes. Testes from 10 controls, 3 heterozygotes, and 3 of each mutant type were analyzed. One entire testis cross-section was scanned for each animal.

The comparison of the stage frequencies was assessed using the synchronization factor as described by van Beek and Meistrich [28], for which we developed a program [29]. Statistical analysis was performed using a one-tailed Student’s *t*-test.

### 2.3. Immunohistochemistry (IHC) and In Situ Hybridization (ISH)

Testes were fixed by intracardiac perfusion of ice-cold 4% (*w*/*v*) paraformaldehyde dissolved in phosphate-buffered saline (PBS) and then kept in the same fixative overnight at 4 °C, washed in PBS, dehydrated, and embedded in paraffin. Sections (5 µm thick) were stored at 4 °C. To assess the specificity of the anti-RARA antibodies or the antisense *Rara* probe, testes from *Rara*^Ser−/−^ and/or *Rara*-knockout mutants [12,19] were used as “negative” controls.

For IHC, sections were processed for antigen retrieval for 20 min at 120 °C in a pressure cooker. They were rinsed in PBS and then incubated in a humidified chamber for 16 h at 4 °C, with the primary antibodies diluted in PBS containing 0.1% (*v*/*v*) Tween 20 (PBST). After rinsing in PBST (three times for 3 min each), detection of the bound primary antibodies was performed for 45 min at room temperature in a humidified chamber using dye-conjugated secondary antibodies. The sections were then counterstained with 4′,6-diamidino-2-phenylindole (DAPI) to label nuclei and with Alexa Fluor 488-conjugated peanut agglutinin (lectin from *Arachis hypogaea*) to label the acrosomal systems. Antibodies used in the IHC, working dilution, and related procedure for antigen retrieval are listed in Table 2. Testes from 4 mice per genotype were analyzed at each time point (PN15, PN21, and 4 months old). IHC experiments were performed in triplicate.

For ISH, the BaseScope v2–RED Reagent kit was used according to the manufacturer’s instructions (Advanced Cell Diagnostics, ref. 323900). Briefly, deparaffinized sections were treated with hydrogen peroxide for 10 min, washed in distilled water, and boiled at 100 °C for 10 min in the target retrieval reagent. Protease IV was then applied for 15 min at 40 °C on dehydrated sections. After rinsing in distilled water, prewarmed probes (Mm-*Ppib*, ref. 320881, and Mm-*Rara*, ref. 824731) were applied on the sections for 2 h at 40 °C. The slides were then washed in the appropriate buffer and subjected to a series of signal amplifications (AMP1 to AMP8). Hybridization signals were detected using the chromogenic Fast RED-B/Fast RED-A reagent. The sections were counterstained for 3 min with 12.5% (*v*/*v*) Harris hematoxylin, and then for 20 s in 0.02% (*v*/*v*) ammonium hydroxide, both diluted in distilled water. Four samples per genotype at PN15 were analyzed in duplicate.

### 2.4. Counts of Sertoli Cells Expressing RARA

Sertoli cell counts were performed on at least 10 seminiferous tubule cross-sections from the testes of PN21 TAM-treated mice (bearing and free of *Tg(Sox9-cre/ER^T2^)*^1Msan^ transgene). Testes from 4 mice per genotype at PN21 were analyzed. The proportion of RARA-expressing Sertoli cells was expressed as a percentage of GATA4-positive Sertoli cells. Statistical analysis was performed using a one-tailed Student’s *t*-test, having verified for equal variance after arcsine transformation of the percentages.

## 3. Results and Discussion

### 3.1. Spermatogenesis Is Not Altered in Males Lacking RARA in Germ Cells

**Figure 1:**
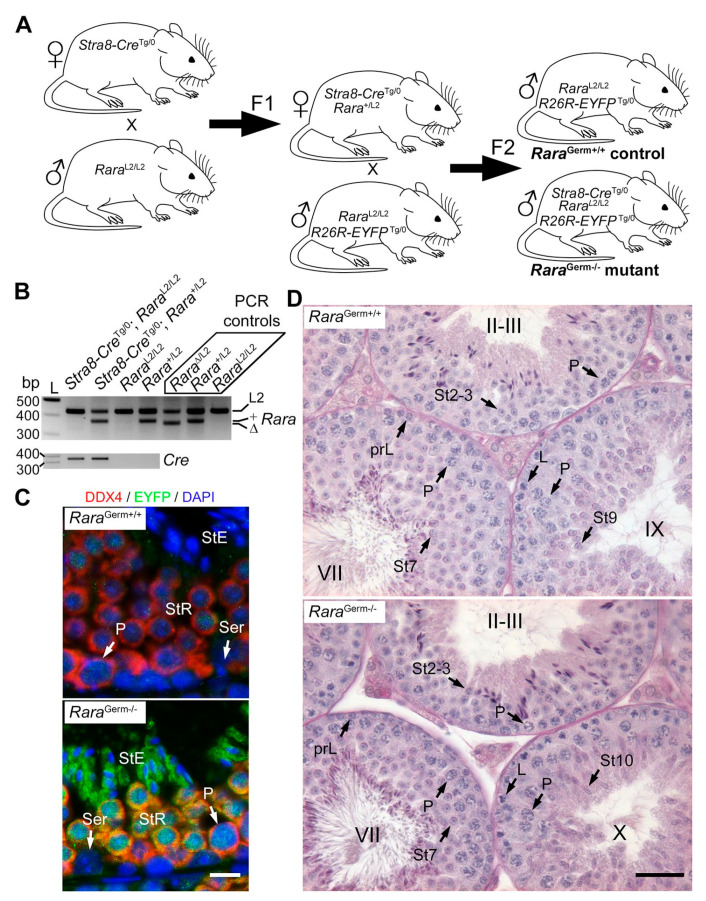
*Rara*^Germ−/−^ mutants display normal spermatogenesis. (**A**) Breeding scheme. *Stra8-Cre*^Tg/0^ females were crossed with *Rara*^L2/L2^ males. This produced *Stra8-Cre*^Tg/0^/*Rara*^+/L2^ F1 females, who were bred with *Rara*^L2/L2^/*R26R-EYFP*^Tg/0^ males to produce control (*Rara*^Germ+/+^) and mutant (*Rara*^Germ−/−^) mice in F2. (**B**) Representative PCR analysis of tail DNA from F2 males with the genotype as indicated. The 427-, 371-, and 357-bp-long fragments correspond to floxed (L2), wild-type (+), and excised (∆) alleles of the *Rara* gene, respectively (upper panel). *Rara*^∆/L2^, *Rara*^+/L2^, and *Rara*^L2/L2^ were used as PCR controls, as indicated. Note that the ∆ allele of *Rara* is absent from the F2 progeny, as expected (upper panel). The 350-bp-long fragment corresponds to the *Tg(Stra8-cre)1Reb* transgene (*Cre*, lower panel). L: ladder; bp: base pair. (**C**) Detection of DDX4 (red signal) and EYFP (green signal) on histological sections of testes from 3-month-old control (*Rara*^Germ+/+^) and mutant (*Rara*^Germ−/−^) mice bearing the reporter transgene. Nuclei were counterstained with DAPI (blue signal). In the control testis, EYFP was not detected (upper panel). In the mutant testis, EYFP was detected in all pachytene spermatocytes and spermatids, but not in Sertoli cells (lower panel). This illustrates efficient Cre-mediated excision in mutant mice. (**D**) PAS stains illustrating normal germ cell associations in the seminiferous epithelium of 3-month-old control (*Rara*^Germ+/+^) and mutant (*Rara*^Germ−/−^) testes, as indicated. Legend: prL, L, and P, preleptotene, leptotene, and pachytene spermatocytes, respectively; Ser, Sertoli cell; St2–3 to St10, steps 2–3 to step 10 of spermatid maturation; StE, elongated spermatid; StR, round spermatid; II–III, VII, IX, and X, epithelial stages. Scale bar: 10 μm in (**C**); 70 μm in (**D**).

**Figure 2:**
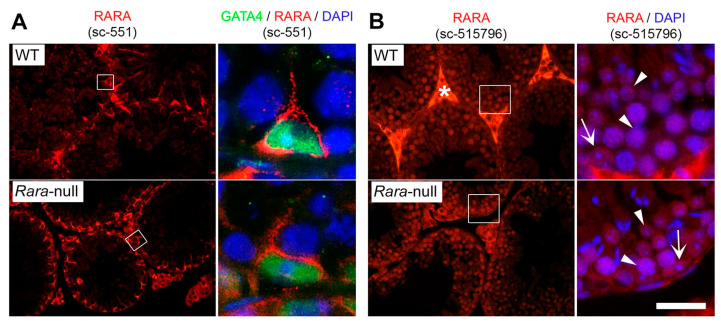
Two commercially available antibodies directed against RARA lacked specificity in IHC experiments. IHC analysis on histological sections of testes from 8-week-old wild-type (WT) and *Rara*-knockout mice (*Rara*-null). (**A**) The sc-551 antibody recognized an epitope (red signal) which was distinct from RARA because it was similarly detected in the cytoplasm of Sertoli cells of WT and *Rara*-null mice. Sertoli cell nuclei were identified by using an antibody directed against the GATA4 transcription factor (green signal). (**B**) The sc-525796 antibody recognized a nuclear epitope (red signal) which was distinct from RARA because it was detected in the nuclei of germ cells (arrowheads) and Sertoli cells (arrows) of WT and *Rara*-null mice. The right panels show enlargements of the boxes in the left panels. Nuclei were counterstained with DAPI (blue signal). Star indicates interstitial signal due to the use of a mouse monoclonal antibody. Scale bar: 80 µm for left panels; 20 μm for right panels.

In the first approach (Figure 1A), we bred *Stra8-Cre*^Tg/0^ females with *Rara*^L2/L2^ males. Then, *Stra8-Cre*^Tg/0^/*Rara*^+/L2^ females were mated with *Rara*^L2/L2^/*R26R-EYFP*^Tg/0^ males to generate the F2 offspring. This was performed because the *Tg(Stra8-cre)1Reb* transgene is not functional in the ovary [20], thereby avoiding deletion of the floxed alleles in the germ cells and transmission of null, excised (∆) alleles of *Rara* to the F2 offspring [31]. Genotyping of these F2 mice showed that the L2 alleles were detected in DNA extracted from tail biopsies of *Tg(Stra8-cre)1Reb*-positive mice, while the excised (∆) allele of *Rara* was not detected, as expected (Figure 1B). Loss of RARA in germ cells could not be evidenced by IHC because (i) all the commercially available antibodies directed against RARA that we tested lack specificity (Figure 2 and Figure S1) and (ii) the RPalpha(F) antibody [30] displays low sensitivity and did not allow for detecting RARA in germ cells [8]. Thus, Cre-mediated ablation of the *Rara* gene in germ cells of 3-month-old males was assessed by visualizing the expression of the excised *Gt(ROSA)26Sor^tm1(EYFP)Cos^* reporter transgene on testis sections. In agreement with previous reports [2,16,17], we found that EYFP was robustly expressed in all germ cells of *Stra8-Cre*^Tg/0^/*Rara*^L2/L2^/*R26R-EYFP*^Tg/0^ males, from the pachytene spermatocyte to the elongated spermatid stages. Expression of EYFP was weaker in the earlier stages of germ cell differentiation (Figure 1C). Moreover, EYFP was not detected in somatic cells, as expected. Assuming that the expression of the reporter faithfully reflects the excision of *Rara* alleles, loss of RARA occurred in all germ cells. Our breading procedure yielded both control (*Rara*^L2/L2^/*R26R-EYFP*^Tg/0^) and mutant (*Stra8-Cre*^Tg/0^/*Rara*^L2/L2^*/R26R-EYFP*^Tg/0^) males, hereafter referred to as *Rara*^Germ+/+^ and *Rara*^Germ−/−^ mice, respectively.

**Figure 3:**
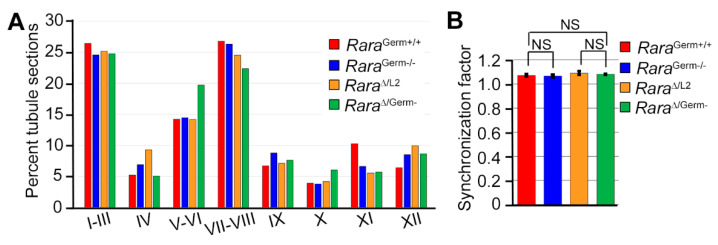
The distribution of the stages of the seminiferous epithelium cycle was normal in mutants lacking RARA in germ cells. (**A**) Relative frequencies of the 12 stages of the seminiferous epithelium cycle (I to XII) in control (*Rara*^Germ+/+^, red bars), heterozygote (*Rara*^∆/L2^, orange bars), and mutant (*Rara*^Germ−/−^, blue bars; *Rara*^∆/Germ−^, green bars) mice. (**B**) Calculated synchronization factors. Error bars represent standard deviations (n = 10 for *Rara*^Germ+/+^; n = 3 for other genotypes). NS, not statistically significant.

Quite surprisingly, in view of the pathological phenotype described in *Rara*-cKO mutants [16], *Rara*^Germ−/−^ males (n = 3) were fertile. When mated with wild-type females, they sired 10 litters for a total of 78 pups. In comparison, *Rara*^Germ+/+^ males (n = 11) sired 21 litters for a total of 146 pups. This was not statistically different from the reproductive capabilities of the *Rara*^Germ−/−^ males (*p* = 0.25). Testis sections of 3-month-old *Rara*^Germ+/+^ (n = 10) and *Rara*^Germ−/−^ males (n = 3) were stained using PAS and analyzed. In both *Rara*^Germ+/+^ and *Rara*^Germ−/−^ males, the cellular associations characterizing the twelve stages of the seminiferous epithelium cycle (I to XII) were readily identified. Moreover, in agreement with the fertility of *Rara*^Germ−/−^ males, their seminiferous epithelium also displayed normal histology (Figure 1D), with the twelve stages of the cycle present in normal proportions (Figure 3A). Accordingly, the mean synchronization factors were similar (Figure 3B). These data indicate that *Rara*^Germ−/−^ males display normal spermatogenesis, as opposed to *Rara*-cKO mutants [16].

**Figure 4:**
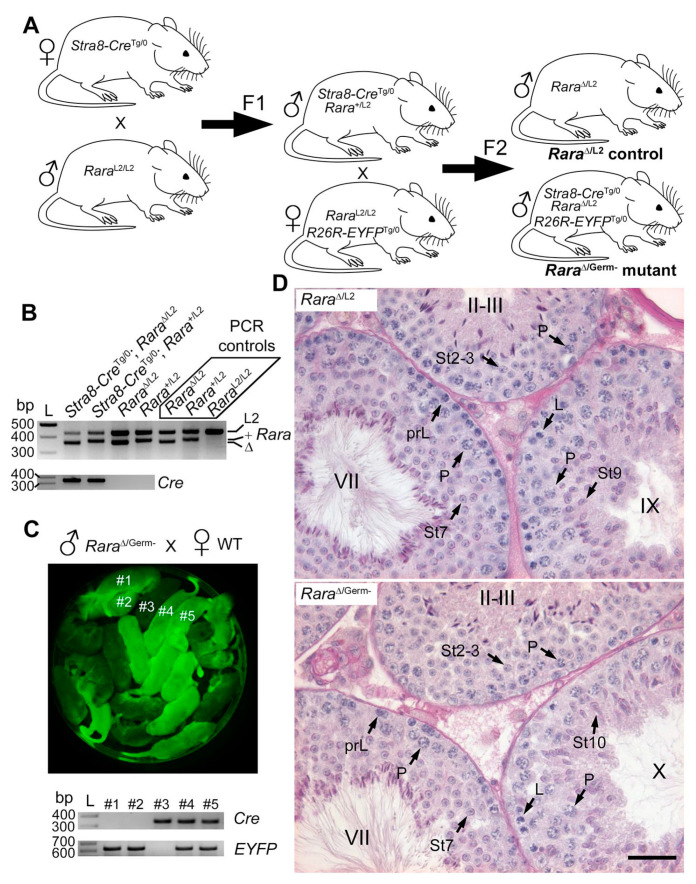
*Rara*^∆/Germ−^ mice display normal spermatogenesis (**A**) Breeding scheme. *Stra8-Cre*^Tg/0^ females were crossed with *Rara*^L2/L2^ males. This produced *Stra8-Cre*^Tg/0^/*Rara*^+/L2^ F1 males, who transmitted either the *Rara*^+^ or the *Rara*^∆^ allele to their F2 progeny when bred with *Rara*^L2/L2^/*R26R-EYFP*^Tg/0^ females. This generated control (*Rara*^∆/L2^) and mutant (*Rara*^∆/Germ−^) mice in F2. (**B**) Representative PCR analysis of tail DNA from F2 males with the genotypes as indicated. The 427-, 371-, and 357-bp-long fragments correspond to floxed (L2), wild-type (+), and excised (∆) alleles of the *Rara* gene, respectively (upper panel). *Rara*^∆/L2^, *Rara*^+/L2^, and *Rara*^L2/L2^ were used as PCR controls, as indicated. An L2 allele, transmitted by the female, was detected in each F2 progeny. The + and ∆ alleles, transmitted by the male, were detected in a mutually exclusive manner. The 350-bp-long fragment corresponds to the *Tg(Stra8-cre)1Reb* transgene (*Cre*, lower panel). L: ladder; bp: base pair. (**C**) Detection of EYFP by ultraviolet light illumination in pups born from a *Rara*^∆/Germ−^ male mated with a wild-type (WT) female (upper panel). EYFP-positive (e.g., #1, #2, #4, and #5) and EYFP-negative (e.g., #3) progenies were obtained in a Mendelian proportion (50%), as expected if almost 100% of germ cells from the father underwent Cre-mediated recombination (i.e., were ablated for the reporter). Representative PCR analysis of tail DNA progenies as indicated. A 350-bp-long fragment corresponds to the *Tg(Stra8-cre)1Reb* transgene (Cre), and a 620-bp-long fragment corresponds to the Cre-recombined reporter (*EYFP*). (**D**) PAS stains illustrating normal germ cell associations in the seminiferous epithelium of 9-month-old control (*Rara*^∆/L2^) and mutant (*Rara*^∆/Germ−^) testes. Legend: prL, L, and P, preleptotene, leptotene, and pachytene spermatocytes, respectively; St2–3 to St10, steps 2–3 to step 10 of spermatid maturation; II–III, VII, IX, and X, epithelial stages. Scale bar: 70 μm in (**D**).

The lack of abnormalities in the testis of *Rara*^Germ−/−^ males compared to *Rara*-cKO males [16] led to questioning the efficacy of *Rara* gene excision in germ cells of *Rara*^Germ−/−^ males [31]. Since the recombination of a given floxed sequence by Cre depends on its genomic context [32], one could figure out that the *Gt(ROSA)26Sor^tm1(EYFP)Cos^* transgene was accessible for Cre-mediated recombination, while the *Rara* locus was not. Thus, in the second approach (Figure 4A), we bred *Stra8-Cre*^Tg/0^/*Rara*^+/L2^ F1 males with *Rara*^L2/L2^/*R26R-EYFP^Tg/0^* females in order to transmit one excised (∆) allele of *Rara* to the F2 offspring. In fact, the sperm of *Stra8-Cre*^Tg/0^/*Rara*^+/L2^ F1 males transmitted either the *Rara*^+^ or the *Rara*^∆^ allele because of the expression of Cre in their germ cells thanks to the *Tg(Stra8-cre)1Reb* transgene. This breeding scheme reduced the amount of floxed *Rara* alleles that Cre had to excise in the germ cells of F2 males and additionally presented the advantage of being identical to the one set up by Peer et al. in their study [16]. Genotyping of tail biopsies from F2 males showed that the excised (∆) allele of *Rara* was present in some animals, even in absence of the *Tg(Stra8-cre)1Reb* transgene (Figure 4B). This observation confirmed that excision of *Rara* had occurred in the sperm of the *Stra8-Cre*^Tg/0^*/Rara*^+/L2^ F1 father, who transmitted a null (∆) allele to its progeny. The *Stra8-Cre*^Tg/0^/*Rara*^∆/L2^/*R26R-EYFP*^Tg/0^ F2 males (hereafter named *Rara*^Δ/Germ−^) were considered mutants (i.e., lacking RARA in germ cells). Importantly, F2 males without the *Tg(Stra8-cre)1Reb* transgene were obtained from the same breeding pairs and were used as controls (hereafter named *Rara*^∆/L2^ males).

Here again, *Rara*^Δ/Germ−^ males (n = 5) were fertile. When mated with wild-type females, they sired 14 litters, with an average of 7.6 pups per litter, a proportion similar to the one obtained from control males (see above). Most importantly, out of the 185 pups born from this breeding, 179 (i.e., ~94%) were *Rara*^∆/+^ and 6 (i.e., 6%) were *Rara*^L2/+^. This finding indicates that recombination of the *Rara* locus by Cre was efficient in almost 94% of the germ cells produced by *Rara*^Δ/Germ−^ males. In agreement with this finding, a Mendelian proportion of pups sired by *Rara*^Δ/Germ−^ males were EYFP-positive when observed under ultraviolet light illumination (Figure 4C). Thus, even though we could not evidence RARA loss by IHC (Figure 2 and Figure S1), we concluded that *Rara*^Δ/Germ−^ mice were actually deprived of RARA in 94% of their germ cells. Next, we carefully analyzed the histology of the testes from 9-month-old *Rara*^Δ/Germ−^ males (n = 3) (Figure 4D). The full representation of the germ cell associations that are characteristic of the 12 stages of the seminiferous epithelium cycle was present in similar proportions in testes from *Rara*^∆/Germ−^ and *Rara*^∆/L2^ males (Figure 3A). Their mean synchronization factor was accordingly similar to that of controls (Figure 3B). Altogether, these experiments show that *Rara* is efficiently inactivated in germ cells without causing any defect in spermatogenesis.

This finding clearly contradicts the proposal that RARA plays an essential role in germ cells [16]. While looking for an explanation for such a discrepancy, we noticed that the *lox*P-flanked allele of *Rara* (*Rara^fl^* allele), used to generate *Rara*-cKO males, still contains a neomycin resistance mini-gene in intron #3, the orientation of which is opposite to that of the *Rara* gene [16]. Often, the insertion of a mini-gene in an intron interferes with the normal functioning of the targeted allele, generating a so-called “hypomorphic” allele driving diminished gene expression (reviewed in [33,34]). One can easily conceive that the expression of *Rara* driven by the *Rara^fl^* allele is not optimal, a possibility that cannot be assessed because males without *Rara^fl^* alleles were used as controls, unfortunately [16]. If the *Rara^fl^* allele is actually hypomorphic, one can hypothesize that *Rara*-cKO males not only lack RARA in germ cells, but they also suffer from RARA insufficiency in all other cell types, including Sertoli cells. In keeping with this hypothesis, it is worth noting that males lacking RARA in Sertoli cells opportunely display vacuolization of their seminiferous epithelium and sloughing of immature germ cells [12], just like *Rara*-cKO males do [16].

Although the present study shows that ablating solely the *Rara* gene in germ cells has no visible impact on spermatogenesis, it was previously found that the combined ablation of *Rara* and *Rarg* in germ cells yields an arrest of spermatogonia differentiation [17], which is more severe than that observed when solely *Rarg* is ablated [15]. This clearly indicates that RARA partially compensates for the loss of RARG, but it is fully dispensable when RARG is present.

### 3.2. RARA Exerts Most, If Not All, of Its Functions on Spermatogenesis in Postnatal Sertoli Cells

Aside from germ cells, RARA is strongly expressed in Sertoli cells [8,12]. The fate of these cells is established during embryonic development, at the time of sex determination. In the mouse, this occurs at around E12.5 [35]. Then, Sertoli cells proliferate prior to puberty and terminally differentiate to become postmitotic at puberty, at around PN15 [36]. RARA expression in Sertoli cells starts as early as E13.5 [37] and persists throughout adulthood [8]. Ablation of *Rara* in Sertoli cells from E13.5 (*Rara^Ser^*^−/−^ mutants) [12] does not allow for discriminating the functions exerted by RARA during the fetal period and at later stages of development. It was recently proposed that both testosterone production by Leydig cells and proliferation of Sertoli cells are controlled by RARA during the fetal period [37]. As some of the defects displayed by *Rara^Ser^*^−/−^ mutants (e.g., vacuolization of the seminiferous epithelium, loss of germ cells) may have arisen as a consequence of altered Sertoli cell proliferation or reduced testosterone synthesis by Leydig cells during fetal life, determining whether prenatal expression of RARA in Sertoli cell is sufficient to ensure normal spermatogenesis remained an important question.

**Figure 5:**
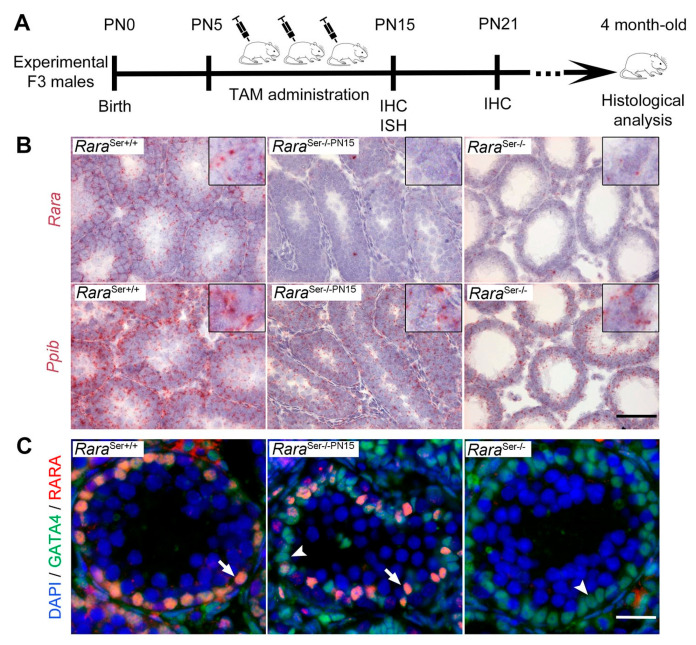
Evidence for ablation of *Rara* gene in postnatal Sertoli cells. (**A**) Diagram illustrating the time-course of TAM administration to F3 *Rara*^L2/L2^ and *Sox9-CreER^T2^* ^Tg/0^/*Rara*^L2/L2^ experimental males (see also Figure S2). Pups were intraperitoneally injected with TAM three times, every other day, between PN5 and PN15 and analyzed at the time points as indicated. (**B**) ISH showing expression of *Rara* and *Ppib* mRNA on testis sections with the indicated genotype at PN15. Nuclei were counterstained with hematoxylin (light blue signal). Few red dots revealing *Rara* mRNA were observed in testis sections from *Rara*^Ser−/−^ and *Rara*^Ser−/−PN15^ mutant males, while numerous red dots were detected in the *Rara*^Ser+/+^ control testis (left panel). As expected, abundant red dots revealing the ubiquitous *Ppib* mRNA were evidenced in each sample. Insets show higher magnifications. (**C**) IHC showing expression of RARA (red signal) and GATA4 (green signal) on testis sections with the indicated genotype at PN15. Overlapping signals yield an orange staining. Nuclei were counterstained with DAPI (blue signal). Arrows and arrowheads point to RARA-positive and RARA-negative Sertoli cell nuclei, respectively. Scale bar: 60 µm in (B); 25 μm in (**C**).

In this context, we aimed to assess the role of RARA in spermatogenesis in postnatal Sertoli cells by inducing ablation of *Rara* using a tamoxifen-inducible Cre-*lox*P mutagenesis system. We chose to ablate the *Rara* gene before Sertoli cells became postmitotic but after the onset of the first spermatogenic wave (i.e., between PN5 and PN15). To do so, *Rara*^L2/L2^ mice were crossed with *Sox9-CreER^T2^* ^Tg/0^ transgenics, thereby generating both *Rara*^L2/L2^ and *Sox9-CreER^T2^* ^Tg/0^*/Rara*^L2/L2^ experimental males from the same breeding pairs (Figure S2A). They were all treated with TAM, as recommended [25], three times between PN5 and PN15 and analyzed at several time points as indicated (Figure 5A).

To evaluate if TAM-treated *Sox9-CreER^T2^* ^Tg/0^*/Rara*^L2/L2^ males actually lacked RARA in their Sertoli cells, we first performed ISH analyses. They showed that *Rara* mRNA could not be detected in their testes (n = 4) at PN15 (i.e., between 4 and 6 days after the last TAM administration). The number of dots detected in this situation was almost identical to that observed in the testes of *Rara*^Ser−/−^ mutants (Figure 5B). Accordingly, TAM-treated *Sox9-CreER^T2^* ^Tg/0^*/Rara*^L2/L2^ males are hereafter referred to as *Rara*^Ser−/−PN15^ mice. Further IHC analyses were performed combining the use of anti-RARA [8,30] and anti-GATA4 antibodies for detecting Sertoli cells. In all the *Rara*^Ser−/−PN15^ mutants analyzed at PN15 (n = 4) and PN21 (n = 4), the seminiferous tubules contained many Sertoli cells devoid of RARA, but some others were still expressing it (Figure 5C and Figure S2B). Quantification on histological sections revealed that 35 ± 11% of Sertoli cells remained RARA-positive at PN21 (Figure S2C). Importantly, these Sertoli cells were always scattered across the seminiferous tubules, with no case where all the Sertoli cells of a given tubule still expressed RARA. Thus, ablation of the *Rara* alleles occurred in about 65% of the Sertoli cells from the *Rara*^Ser−/−PN15^ mutants, and no seminiferous tubule escaped Cre-mediated recombination. The reason RARA remains present in some Sertoli cells is unclear. It is possible that the protein half-life is long enough to make RARA persist for a few hours, even in the absence of its mRNA [38].

**Figure 6:**
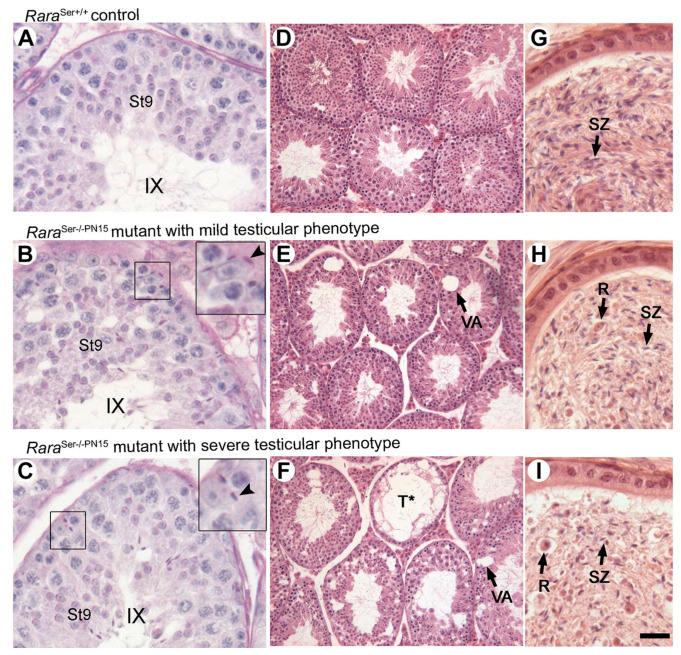
Loss of RARA in Sertoli cells from PN15 yields seminiferous epithelium defects in adulthood. Histological analyses of testis and epididymis of 4-month-old control and *Rara*^Ser−/−PN15^ mice. (**A**–**C**) PAS stains of histological sections through the testis, illustrating retained mature spermatids at epithelial stage IX in *Rara*^Ser−/−PN15^ mutants. The insets show high-magnification views of the boxed areas. Arrowheads point to retained mature spermatids. (**D**–**F**) HE stains of histological sections through the testis, illustrating vacuolation of the seminiferous epithelium in *Rara*^Ser−/−PN15^ mutants. (**G**–**I**) HE stains of histological sections through the caudal epididymis. The epididymis of *Rara*^Ser−/−PN15^ mutants contains fewer spermatozoa (compare SZ in **G**–**I**) but numerous round germ cells. Legend: R, round immature germ cells; St9, step 9 of spermatid maturation; SZ, spermatozoa; T*, tubule depleted of germ cells; VA, vacuoles; IX, epithelial stage. Scale bar: 75 μm in (**A**–**C**) and (**G**–**I**); 150 μm in (**D**–**F**).

Most importantly, despite the persistence of RARA in some Sertoli cells, all the *Rara*^Ser−/−PN15^ mutants were infertile (n = 19). To uncover the reason for their infertility, we performed histological analyses at 3.5 months after the last TAM treatment (Figure 6). Testis sections from these males (n = 12) showed that mature spermatids failed to align at the luminal side of the seminiferous epithelium and were often retained within the epithelium (insets in Figure 6B,C). Many seminiferous tubule sections, distributed through-out the testis, displayed large vacuoles (Figure 6E,F) or were depleted of germ cells (Figure 6F). Accordingly, the caudal epididymis contained few spermatozoa but an unusually high number of round immature germ cells, suggesting extensive desquamation of cells from the seminiferous epithelium (Figure 6H,I). The degree of vacuolation and tubule degeneration varied between *Rara*^Ser−/−PN15^ mutants. About 80% of them (i.e., 10 out of 12) were considered severely affected; more than half of their seminiferous tubule sections contained more than two vacuoles (Figure 6C,F,I). The remaining 20% were mildly affected; about one-fifth of their seminiferous tubules sections contained one or two vacuoles (Figure 6B,E,H). These alterations were not regionalized to a particular area but were distributed across the testes.

Interestingly, the histological abnormalities displayed by our *Rara*^Ser−/−PN15^ mutants are similar in all respects to those we described in *Rara*^Ser−/−^ mice, including the variability between individuals [12]. This finding indicates that (i) heterogeneity in the severity of the abnormalities is not linked to the proportion of Sertoli cells that have lost RARA, and (ii) RARA produced by Sertoli cells until PN15 is dispensable for the proper development of the seminiferous epithelium. In this context, our study casts doubt on the proposal that RARA is instrumental to Sertoli cell proliferation during the fetal period [37].

**Figure 7:**
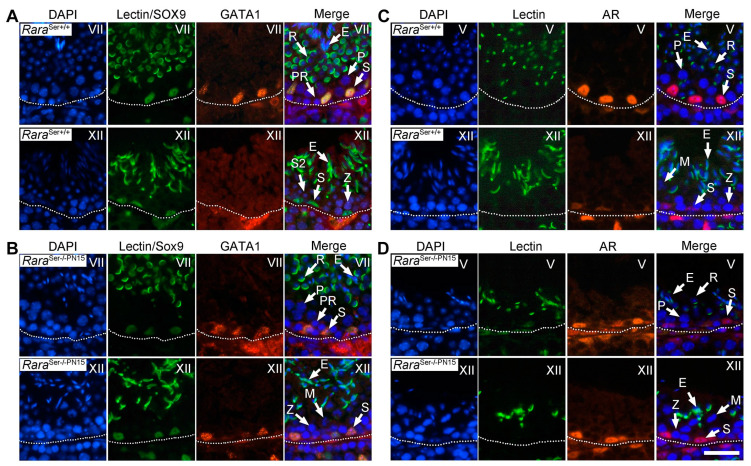
Loss of RARA in Sertoli cells from PN15 abrogates the periodical expression of genes in adulthood. IHC analysis on histological sections from testes of 4-month-old control and *Rara*^Ser−/−PN15^ mutant mice. (**A**,**B**) In the control, GATA1 expression (red signal) was strong in Sertoli cell nuclei at stage VII and at the background level at stage XII of the seminiferous epithelium cycle. In the *Rara*^Ser−/−PN15^ testis, GATA1 was uniformly expressed at all epithelial stages. Sertoli cell nuclei were identified using an antibody directed against SOX9 (green signal in Sertoli cells). (**C**,**D**) In the control, AR expression (red signal) was strong in Sertoli cell nuclei at stage V and weak at stage XII. In the *Rara*^Ser−/−PN15^ testis, AR was uniformly expressed at all epithelial stages. Nuclei were counterstained by DAPI (blue signal). In (**A**–**D**), the acrosomal system is identified by Alexa Fluor 488-conjugated peanut agglutinin (green signal), allowing proper staging. The dotted lines indicate the periphery of seminiferous tubules. Legend: E, elongated spermatids; M, spermatocytes in metaphase; PR and P, preleptotene and pachytene spermatocytes, respectively; R, round spermatids; S, Sertoli cells; S2, type 2 spermatocytes; Z, zygotene spermatocytes. Roman numerals designate stages of the seminiferous epithelium cycle. Scale bar: 70 μm.

Several genes normally display a periodical pattern of expression in Sertoli cells at given stages of the seminiferous epithelium cycle [39,40]. In the present study, we investigated the periodical expression of selected genes in Sertoli cells through IHC analysis of 4-month-old testes using anti-GATA-1 [41] and anti-androgen receptor (AR) [42] antibodies. As expected, GATA1 expression in Sertoli cells was high at stage VII and very low at stage XII of the seminiferous epithelium cycle in control testes (Figure 7A). In *Rara*^Ser−/−PN15^ mutants, GATA1 was detected in Sertoli cells at all stages of the seminiferous epithelium cycle (Figure 7B). Along the same lines, AR expression was robust in Sertoli cells at stage V and weak at stage XII of the control testes (Figure 7C), but it was high all along the cycle of the seminiferous epithelium in *Rara*^Ser−/−PN15^ mutants (Figure 7D). We conclude from these experiments that the loss of RARA in Sertoli cells during puberty abrogates their periodical expression of GATA1 and AR. Interestingly, this occurs even though RARA appears not lost in all Sertoli cells of a given seminiferous tubule. As a similar phenomenon is observed in a germ cell-deficient environment [1], it is possible that abrogation of the periodical expression of genes in Sertoli cells of *Rara*^Ser−/−PN15^ mutants is linked to their loss of germ cells. Our present data thus suggest that RARA maintains the periodical activity of Sertoli cells rather than initiating it during fetal development, as we previously proposed [12].

## 4. Conclusions

RARs are nuclear transcription factors that, once activated by ATRA, regulate the expression of target genes, which in turn control various processes, among which is spermatogenesis. As *Rara*-knockout mice display a pathological testis phenotype and RARA is expressed in both somatic and germ cells of the testis, a strong research effort has been directed towards the identification of the cell types in which RARA is operational [12,13,16,17,43]. Our present study adds to this effort. Firstly, by showing that ablation of RARA in germ cells has no effects on testis integrity and functioning, it invalidates the recent proposal that RARA has dual functions in germ cells, balancing proliferation and differentiation of spermatogonia and controlling genome integrity during meiosis. Secondly, it shows that ablation of RARA in Sertoli cells, starting from PN15, recapitulates the complete set of abnormalities induced by knocking out *Rara* in all cells. Thus, it demonstrates that RARA exerts all its functions in Sertoli cells only, and at puberty but not at fetal stages. Although being a challenging and difficult task, it is now necessary to reveal more specifically the gene networks and mechanisms that RARA governs in Sertoli cells to allow proper spermatogenesis. Animal models with controlled ablation of *Rara* in Sertoli cells as we presented here could be useful in such future studies.

## Figures and Tables

**Table 1:** Sequences of the primers used for genotyping mice.

Gene or Transgene	Forward Primer (5′ to 3′)	Reverse Primer (5′ to 3′)	Amplicon Size (bp)
*Rara* (+ allele) *	CAGGGAGGATGCTGTTTGTA	AACTGCTGCTCTGGGTCTCG	371
*Rara* (L2 allele) *			427
*Rara* (∆ allele) *		TACACTAACTACCCTTGACC	357
*Tg(Stra8-cre)*^1Reb^ and *Tg(Sox9-cre/ER^T2^)*^1Msan^	ATTTGCCTGCATTACCGGTC	ATCAACGTTTTCTTTTCGGA	350
*Gt(ROSA)26Sor*^tm1(EYFP)Cos^ (excised allele)	AAGGGAGCTGCAGTGGAGTA	TGGTGCAGATGAACTTCAGG	620

* +, L2, and Δ correspond to wild-type, floxed, and excised allele of *Rara* gene, respectively.

**Table 2:** List of antibodies used in the present study.

Antigen	Species	Reference	Source	Dilution	Antigen Retrieval	Secondary Antibody
AR	Rabbit	sc-816	Santa Cruz Biotechnologies	1/100	Protocol 1	Cy3-conjugated donkey anti-rabbit IgG
DDX4	Rabbit	ab13840	Abcam	1/1000	Protocol 1	Cy3-conjugated goat anti-rabbit IgG
EYFP	Chicken	GFP-1020	Aves	1/1000	Protocol 1	Alexa Fluor 488-conjugated goat anti-chicken IgG
GATA1	Rat	sc-265	Santa Cruz Biotechnologies	1/50	Protocol 1	Cy3-conjugated donkey anti-rat IgG
GATA4	Goat	sc-1237	Santa Cruz Biotechnologies	1/75	Protocol 1	Alexa Fluor 488-conjugated donkey anti-goat IgG
RARA	Rabbit	RPalpha(F)	[30]	1/50	Protocol 2	Cy3-conjugated donkey anti-rabbit IgG
RARA *	Rabbit	sc-551	Santa Cruz Biotechnologies	1/100	Protocol 1	Cy3-conjugated donkey anti-rabbit IgG
RARA *	Mouse	sc-515796	Santa Cruz Biotechnologies	1/25	Protocol 1	Cy3-conjugated donkey anti-mouse IgG
RARA **	Mouse	NB200-322	NovusBio	1/1000	Protocol 1	Cy3-conjugated donkey anti-mouse IgG
RARA **	Rabbit	NBP2-58082	NovusBio	1/100	Protocol 2	Cy3-conjugated donkey anti-rabbit IgG
RARA **	Rabbit	CS-155-100	Diagenode	1/1000	Protocol 2	Cy3-conjugated donkey anti-rabbit IgG
RARA **	Rabbit	C15310155	Diagenode	1/500	Protocol 3	Cy3-conjugated donkey anti-rabbit IgG
SOX9	Rabbit	AB5535	Merk Millipore	1/1000	Protocol 1	Alexa Fluor 488-conjugated donkey anti-rabbit IgG

Protocol 1: 10 mM sodium citrate buffer (pH = 6.0); Protocol 2: 10 mM tris base, 1 mM EDTA, and 0.05% (*v*/*v*) Tween 20 (pH = 9.0); Protocol 3: 0.05% citraconic anhydride buffer (pH = 7.4). * These antibodies recognize epitopes which are not RARA (see below); ** these antibodies recognize epitopes which are not RARA, as illustrated in Figure S1.

## Data Availability

The authors confirm that the data supporting the findings of this study are available within the article and its Supplementary Materials.

## Supplementary Materials

The following supporting information can be downloaded at: https://www.mdpi.com/article/10.3390/cells11050891/s1. Figure S1: Four commercially available antibodies directed against RARA recognize epitopes which are not RARA and are therefore not suitable for IHC experiments. Figure S2: Generation of mice in which *Rara* deletion is induced in Sertoli cells after birth, and quantitative assessment of RARA loss.

## Abbreviations

ATRA	All-*trans* retinoic acid
DAPI	4′,6-diamidino-2-phenylindole
E13.5	Embryonic day 13.5
HE	Hematoxylin–eosin
IHC	Immunohistochemistry
ISH	In situ hybridization
PAS	Periodic acid–Schiff
PBS	Phosphate-buffered saline
PBST	Phosphate-buffered saline containing Tween 20
PN0 to PN21	Postnatal days 0 to 21
RAR	Retinoic acid receptor
TAM	Tamoxifen
