# Supplementary material for: Retinoic Acid Receptor Alpha Is Essential in Postnatal Sertoli Cells but Not in Germ Cells
Source: Cells. 2022 Mar 4;11(5):891. doi: 10.3390/cells11050891 (PMC8909012; doi:10.3390/cells11050891)
Supplement: Supplementary file 1 [file cells-11-00891-s001.zip › cells-1603595-supplementary.pdf]

cells

MDPI

Article ***Condrea D. et al., Cells 2022**, 11*

Retinoic Acid Receptor Alpha Is Essential in Postnatal Sertoli Cells, but Not in Germ Cells

Diana Condrea [1,†], Sirine Souali-Crespo [1,†] , Betty Féret [1], Muriel Klopfenstein [1], Sylvain Faisan [2], Manuel Mark [1,3], Norbert B. Ghyselinck [1], Nadège Vernet [1]

[1] Institut de Génétique et de Biologie Moléculaire et Cellulaire (IGBMC), Département de Génétique Fonctionnelle et Cancer, Centre National de la Recherche Scientifique (CNRS, UMR7104), Institut National de la Santé et de la Recherche Médicale (INSERM U1258), Université de Strasbourg (UNISTRA), 1 rue Laurent Fries, CEDEX, 67404 Illkirch, France

[2] ICube (UMR 7357), Laboratoire des Sciences de l'Ingénieur, de l'Informatique et de l'Imagerie, UNISTRA, CNRS, 300 Boulevard Sébastien Brant, CEDEX, 67412 Illkirch, France

[3] Service de Biologie de la Reproduction, Hôpitaux Universitaires de Strasbourg (HUS), 67000 Strasbourg, France

† These authors contributed equally to this work.

Supplementary Figures

Figure S1. Four commercially available antibodies directed against RARA recognize epitopes which are not RARA, and are therefore not suitable for IHC experiments.

Figure S2. Generation of mice in which *Rara* deletion is induced in Sertoli cells after birth, and quantitative assessment of RARA loss.

cells

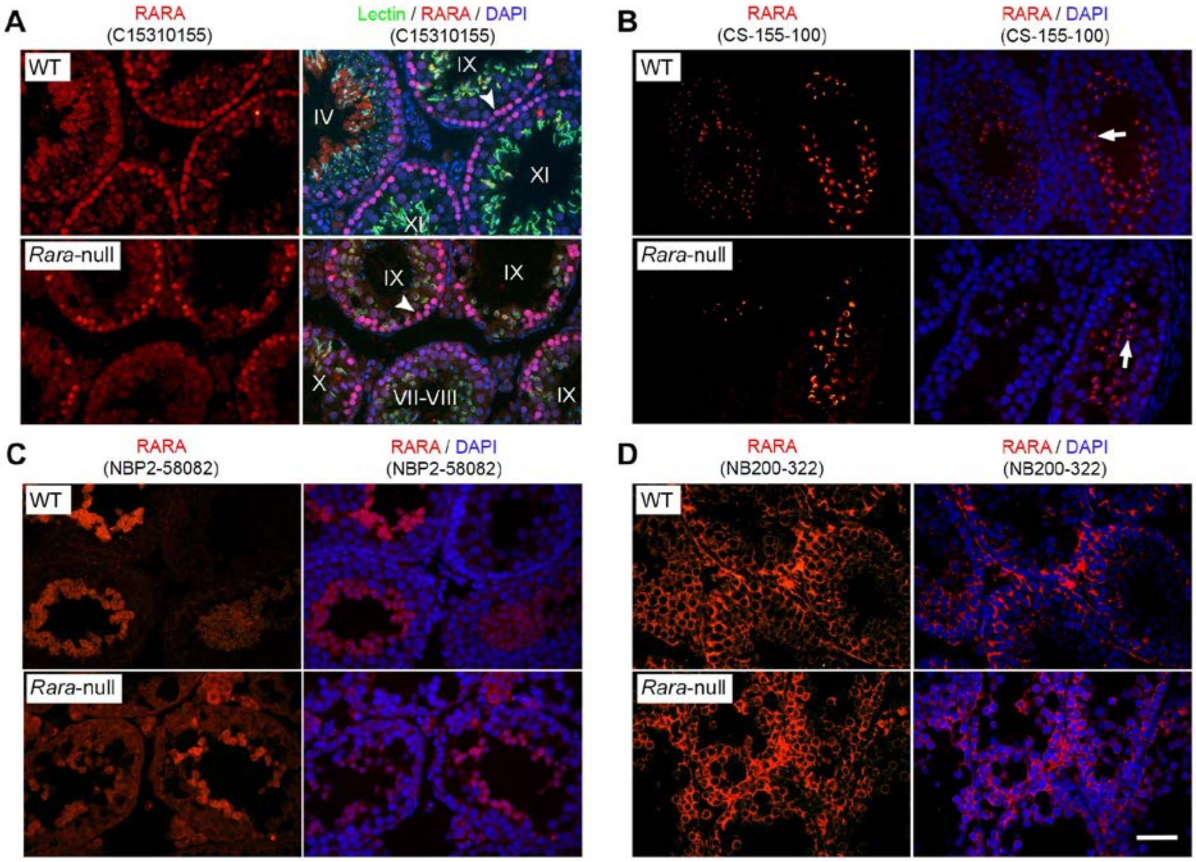

Figure S1. Four commercially available antibodies directed against RARA recognize epitopes which are not RARA, and are therefore not suitable for IHC experiments. IHC on histological sections from testes of 2-month old (A) and 2-week-old (B-D) wild-type (WT) and *Rara*-knockout (*Rara*-null) mice. **(A)** The C15310155 antibody recognizes a nuclear epitope in spermatocytes (red signal), which is not RARA, because it is similarly detected in WT and RARA-deficient mice (arrowheads). The acrosomal system is labeled by AlexaFluor 488-conjugated peanut agglutinin (green signal), allowing proper staging. Roman numerals designate stages of the seminiferous epithelium cycle. **(B)** The CS-155-100 antibody recognizes an acrosome-like perinuclear epitope in spermatids (red signal), which is not RARA, because it is similarly detected in WT and RARA-deficient mice (arrows). **(C)** The NBP2-58082 antibody recognizes an epitope in spermatids (red signal), which is not RARA, because it is similarly detected in WT and *Rara*-null mice. **(D)** The NB200-322 antibody recognizes a cytoplasmic epitope in germ cells (red signal), which is not RARA, because it is similarly detected in WT and *Rara*-null mice. Nuclei were counterstained with DAPI (blue signal). Scale bar in D: 50 µm.

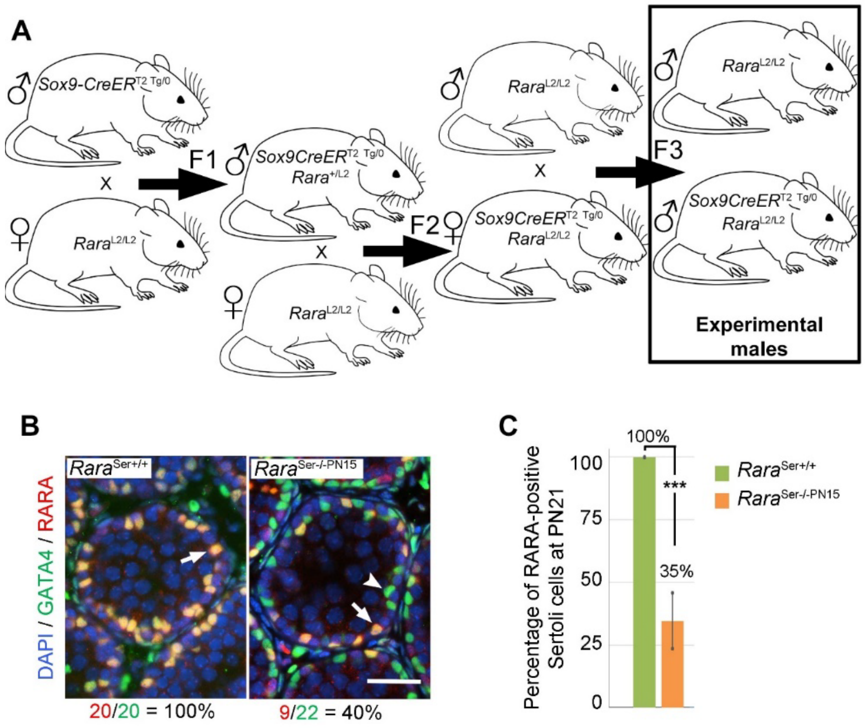

Figure S2. Generation of mice in which *Rara* deletion is induced in Sertoli cells after birth, and quantitative assessment of RARA loss. **(A)** *Sox9-CreER*$^{T2\ Tg/0}$ males were crossed with *Rara*$^{L2/L2}$ females. This produced *Sox9-CreER*$^{T2\ Tg/0}$;*Rara*$^{+/L2}$ F1 males, who were bred with *Rara*$^{L2/L2}$ females to produce *Sox9-CreER*$^{T2\ Tg/0}$; *Rara*$^{L2/L2}$ F2 females. These females were then mated to *Rara*$^{L2/L2}$ males to produce, from the same litters, F3 *Rara*$^{L2/L2}$ (control) and *Sox9-CreER*$^{T2\ Tg/0}$;*Rara*$^{L2/L2}$ (experimental) males, which are being treated by TAM. **(B)** Detection of RARA (red signal) and GATA4 (green signal) on histological sections from TAM-treated control (designated as *Rara*$^{Ser+/+}$) and experimental (designated as *Rara*$^{Ser-/-PN15}$) males at PN21. Overlapping signals yield an orange staining. Nuclei were counterstained with DAPI (blue signal). Arrows and arrowheads point to RARA-positive and RARA-negative Sertoli cell nuclei, respectively. Scale bar (in) B: 70 µm. **(C)** Percentages of RARA-positive Sertoli cells in tubule sections from *Rara*$^{Ser+/+}$ and *Rara*$^{Ser-/-PN15}$ males at PN21. Bars represent mean of n=4 ± SD (***, statistically different with p<0.001).
